# Optical Fiber Magnetic Field Sensors Based on Magnetic Fluid: A Review

**DOI:** 10.3390/s18124325

**Published:** 2018-12-07

**Authors:** Nélia Alberto, Maria Fátima Domingues, Carlos Marques, Paulo André, Paulo Antunes

**Affiliations:** 1 Instituto de Telecomunicações, Campus Universitário de Santiago, 3810-193 Aveiro, Portugal; fatima.domingues@ua.pt (M.F.D.); carlos.marques@ua.pt (C.M.); pantunes@ua.pt (P.A.); 2 Department of Physics & I3N, University of Aveiro, Campus Universitário de Santiago, 3810-193 Aveiro, Portugal; 3 Department of Electrical and Computer Engineering and Instituto de Telecomunicações, Instituto Superior Técnico, University of Lisbon, 1049-001 Lisbon, Portugal; paulo.andre@lx.it.pt

**Keywords:** optical fiber sensors, magnetic field, magnetic fluid, fiber grating, interferometric sensor, surface plasmon resonance (SPR), tailored fibers

## Abstract

Magnetic field sensing is an important issue for many application areas, such as in the military, industry and navigation. The current sensors used to monitor this parameter can be susceptible to electromagnetic interferences, however due to their advantages over the traditional sensors, the optical fiber devices could be an excellent alternative. Furthermore, magnetic fluid (MF) is a new type of functional material which possesses outstanding properties, including Faraday effect, birefringence, tunable refractive index and field dependent transmission. In this paper, the optical fiber magnetic field sensors using MF as sensing element are reviewed. Due to the extensive literature, only the most used sensing configurations are addressed and discussed, which include optical fiber grating, interferometry, surface plasmon resonance (SPR) and other schemes involving tailored (etched, tapered and U-shaped) fibers.

## 1. Introduction

Magnetic field sensors have been widely used in many scientific and industrial applications, including biomedical detection, the aviation industry, space and geophysical research, and controlled nuclear fusion [1,2,3,4]. The traditional methods often use the Hall effect, magneto-transistor, magnetodiode, fluxgate, magneto-resistive (amorphous and giant magneto-resistors) or other semiconductor effects to sense magnetic fields [5,6,7,8,9,10]. Nevertheless, these devices present some disadvantages related to their miniaturization, power consumption, cost, lack of stability, reduced multiplexing capability and remote monitoring. Another relevant drawback is the inherent sensitivity to several environmental magnetic field sources, which induces measurements’ noise. In addition, the metallic circuits and signal transmissions cables are also susceptible to electromagnetic interference [9,10,11]. 

When comparing these sensing technologies with optical fiber sensors, the latter has received special attention in the last years, since they offer several outstanding advantages over their electronic counterparts. Among other characteristics, it can be highlighted that the immunity to electromagnetic interference, small size, remote sensing capabilities, and resistance in hazardous environments, make this technology suitable to be used in severe conditions, such as gas pipelines or electrically sensitive environments.

Currently, several optical fiber sensors for magnetic field monitoring have been reported, based on different sensing techniques, including the Faraday effect [12,13] and magnetostrictive materials [14,15,16]. Nevertheless, in certain cases, the integration process of this type of material with the optical fiber is not easy. As an alternative, there is the magnetic fluid (MF), which has become widely applied as a sensing element for magnetic field detection. Due to its versatile magneto-optical properties, this attractive material has been used in distinct MF-based optical devices, such as modulators [17], optical switchers [18], couplers [19], optical gratings [20] and also as magnetic field sensors [21,22]. However, in addition to all these advantages, this material also presents some drawbacks when applied in a sensor context. Among them, as further discussed in the following sections, it can be referred the hysteresis time of the MF and the dependency of the MF’s refractive index with the temperature. In the case of the first effect, the delay time is assigned to the MF’s viscosity [23]. Due to oscillations, the magnetic particles take a certain time to keep balance. Although this problem cannot be eliminated, this could be reduced if the magnetic field changes slowly. The second effect involves, in some cases, the use of additional schemes to compensate for the thermal influence. Despite these two aspects, the number of the MF-based magnetic field sensors is increasing, reflecting the advantageous characteristics of these materials.

Optical fiber sensors can be classified depending on which property is being considered, for instance, modulation process, working principle, application field, etc. Being aware of the impossibility to address all the categories and discuss all the works already published, this work intends to present a general revision and discussion on the optical fiber magnetic field sensors based on MF. Here, we consider three different classes of optical fiber sensors, namely grating-based sensors, interferometric sensors, and sensors based on other sensing schemes, according with Table 1. The combination of optical fibers and MF can occur in three different ways, as MF film, the filling material, and the cladding of a tapered/etched fiber, for instance. 

The document is structured in the following sections: following this introductory section (Section 1), a brief description of the behavior of MF under magnetic field is presented (Section 2). The next sections are focused on the discussion of magnetic field sensors based on different sensing principles (Section 3, Section 4 and Section 5). The final remarks of this review are summarized in the Section 6. The latter contains a table (Table 2) where the main sensing parameters of the sensors are summarized. To simplify the comparison process of the sensors’ performance, the units were standardized. It should be noted that throughout the manuscript, the data is presented in the units in which the works were published, in order to be consistent with the presented figures. Thus, with respect to the magnetic field, although most of the data are expressed in militesla (mT), it will be found other units, including Oersteds (Oe) and Gauss (G). For the knowledge, the conversion is as follow: 1 Oe = 1 G = 0.1 mT. Also, due to the high number of acronyms used in this article, at the end of the manuscript, a list is included as an Appendix.

## 2. Magnetic Fluid 

MF is a type of stable colloid usually constituted by magnetic nanoparticles (i.e., Fe_3_O_4_) with surfactant, highly dispersed in a liquid carrier, usually an organic solvent or water. MF possesses numerous interesting optical properties, including tunable refractive index, tunable transmittance, birefringence and the Faraday effect, among others [24,25]. 

**Figure 1:**
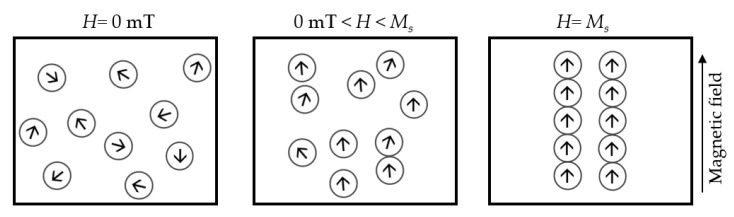
The behavior of the MF nanoparticles under different magnetic field intensities.

When exposed to an external magnetic field, the structural pattern state of the MF changes from randomly homogenous to a field-dependent structural pattern. The nanoparticles cluster and form chains and columns along the magnetic field direction (see Figure 1), resulting in changes of the MF’s refractive index [26,27]. When all the nanoparticles are gathered and aligned in the magnetic chains, at the saturation magnetization (*M_s_*) level of the magnetic field (*H*), the MF’s refractive index will become unchangeable. 

The thermal and the magnetic energies of the nanoparticles are the two main physical energies associated to the organization of the magnetic particles in columns, when the MF is under an external *H*. The dependence of the refractive index of the MF (*n_MF_*) with the *H* and temperature (*T*) can be described with the Langevin function [28]:

(1) $$n_{MF}=\;\left(n_{s}-n_{0}\right)\left[coth\left(\propto \frac{H-H_{c}}{T}\right)-\frac{T}{\propto \left(H-\;H_{c}\right)}\right]+n_{0},\;\;\mathrm{for}\;\;H>H_{c}$$

where *n_s_* is the saturated value of the refractive index of the MF and *n*_0_ is the refractive index of the MF under *H* lower than *H_c_*. *H_c_* denotes the critical *H* when the *n_MF_* starts to change, which depends of the carrier liquid type and the concentration of the MF. *H* is the magnetic field in Oersteds, *T* is the temperature of the fluid in Kelvin and *α* represents the fitting parameter. 

Since the magnetoelectric effect occurs when external *H* is acting on the MF, the electric susceptibility (*χ*) changes, resulting in the variation of the *n_MF_*. The relation between these two parameters is given by [29]:

(2) $$n_{MF}=\sqrt{\varepsilon_{MF}}=\;\sqrt{1+\chi }$$

where *ε_MF_* is the dielectric constant of the MF. 

The electric susceptibility of the MF is also dependent on the intensity of the *H* and on the relative direction between the electric field (*E*) and the *H* [30,31]:

(a) If *E* is perpendicular to *H*, $\frac{\partial \chi }{\partial H}<0$, then the *n_MF_* will decrease with the magnetic field;
(b) If *E* is parallel to *H*, $\frac{\partial \chi }{\partial H}>0$, then the *n_MF_* will increase with the magnetic field.

## 3. Grating-Based Sensors

### 3.1. FBG-Based Sensors

**Figure 2:**
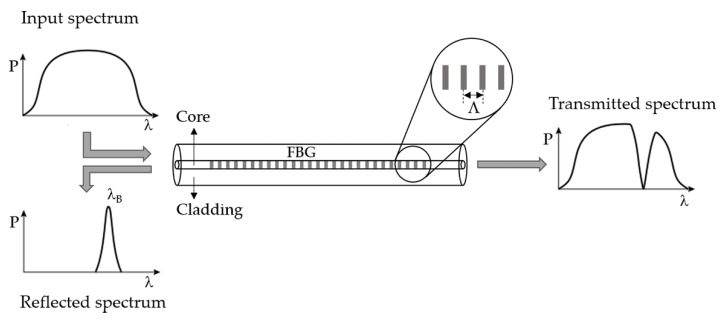
Schematic representation of the working principle of an FBG.

A FBG consists in a periodic modulation of the refractive index of the optical fiber core, normally produced by exposing the optical fiber to an optical pattern of ultraviolet (UV) interference. This periodic modulation acts as a selective filter for the wavelengths that satisfy the Bragg condition, transmitting all the others. The Bragg condition is given by the following equation:

(3) $$\lambda_{B}=2n_{eff}\Lambda$$

where *λ_B_* represents the reflected Bragg wavelength, *n_eff_* is the effective refractive index of the propagate core mode and *Λ* is the grating period. Figure 2 shows a schematic representation of the working principle of an FBG [32].

In common conditions, the FBG response is not influenced by the external refractive index, however if the fiber cladding diameter is reduced along the grating region, the *n_eff_* is affected by these variations. 

Using etched FBG (eFBG) as sensing element, Dai et al. proposed a sensor for magnetic field up to 25 mT, which was applied perpendicular to the axial of the FBG [33]. A nanosized Fe_3_O_4_ MF, previously prepared by the chemical co-precipitation method, was injected into micro-tubes containing fibers with different diameters, namely 11.3, 10.0 and 8.5 μm. As predicted through the theoretical simulation, the results reveal a nonlinear dependence of the wavelength shift of the FBGs with the magnetic field, being the fiber with the smaller diameter the sensor with greater sensitivity (wavelength shift of 86 pm when the magnetic field increases to 25 mT). The response time is about 15 s. The sensor only shows reverse response for a magnetic field up to 16 mT. For higher magnetic field values, the electrostatic repulsion and Brownian motion are insufficient to eliminate the adhesion force between ferromagnetic particles and magnetic chains. 

**Figure 3:**
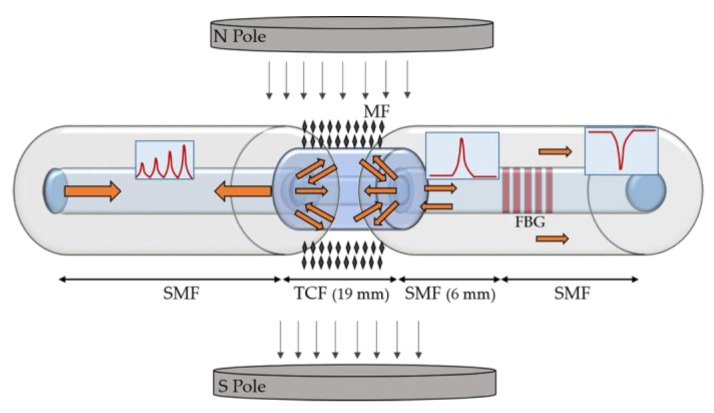
Schematic representation of the TCF-FBG based magnetic field sensor (adapted from [34]).

To overcome the mechanical fragility and low reflectivity presented by an eFBG-based solution, and additionally the cross-sensitivity to the temperature, which influences the accuracy on the magnetic field detection, Tian et al. proposed a temperature-independent magnetic field sensor [34]. The design used, based on a thin core fiber (TCF) sandwiched in the upstream of an FBG, is schematically shown in Figure 3. The magnetic field is obtained from the variation of the optical power of the cladding mode resonances, and the temperature is determined by the core mode wavelength shift. Two permanent magnets were used to generate a uniform magnetic field in the range of 0 to 17 mT, perpendicularly to the TCF-FBG structure, which was placed into a glass capillary tube filled with a highly stable aqueous solution of ferromagnetic nanoparticles. The proposed sensor presents a sensitivity of –0.78 dB/mT in the range of 7 to 15 mT, a thermal sensitivity of 0.068 dB/°C and a response time of about 30 s. When compared with the previous technique, the main advantages of this solution is the use of the power-interrogation, which decreases the data acquisition costs, and the low temperature sensitivity. 

The sensor proposed by Yang et al. consists of an FBG cascaded by a 2 cm-cleaved optical fiber end, which was surrounded by EMG 607 MF [35]. The sensing principle is based on Fresnel reflectivity changes at the fiber end face when the magnetic field varies, as result of the MF’s refractive index alteration. By measuring the reflectivity ratio between the FBG reflection peak and the reflection background, it is possible to monitor magnetic fields in the range from 0 to 50 Oe. Furthermore, the temperature can be measured through the Bragg wavelength monitoring. The proposed device is characterized by high accuracy and stability, since the influence of the power level fluctuation of the optical source in the results is eliminated. 

### 3.2. TFBG-Based Sensors

TFBG is a type of short-period grating, in which the grating planes are tilted with respect to the fiber axis [36]. The tilted angle of the grating plane enhances the light coupling from the core mode to the cladding modes. This particularity makes these structures more advantageous than uniform FBGs for sensing applications, since the cladding mode resonances in a TFBG are sensitivity to parameters such as the surrounding refractive index.

**Figure 4:**
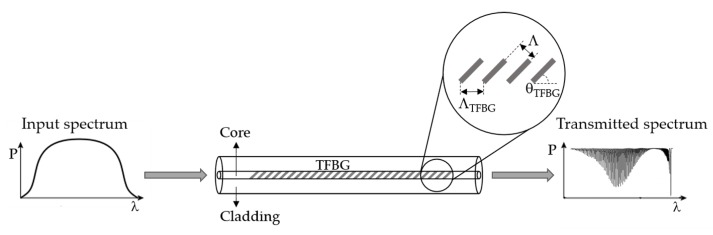
Schematic representation of a TFBG.

Figure 4 shows a schematic representation of a TFBG. The transmission spectrum contains the core mode (longest wavelength) and a set of resonances at shorter wavelengths, which correspond to the cladding modes. Between them, there is the ghost mode, which is a cladding mode that interacts weakly with the cladding boundary. 

The resonance wavelength of the core mode (*λ*_TFBG_) and the *i^th^* cladding mode ($\lambda_{clad}^{i}$) are determined by the phase-matching condition, which can be expressed as:

(4) $$\lambda_{TFBG}=\frac{2n_{eff,core}\Lambda }{cos\theta_{TFBG}}\;\mathrm{and}\;\;\;\lambda_{clad}^{i}=\frac{\left(n_{eff,core}^{i}+n_{eff,clad}^{i}\;\right)\Lambda }{cos\theta_{TFBG}}$$

where $n_{eff,core}$ is the refractive index of the core mode at λ_TFBG_. $n_{eff,core}^{i}$ and $n_{eff,clad}^{i}$ are the refractive index of the core mode and the *i^th^* cladding mode at $\lambda_{clad}^{i}$, respectively. $\theta_{TFBG}$ is the tilt angle of the grating planes related to the perpendicular of the fiber axis. Λ corresponds to the nominal grating period which is described as $\Lambda =\Lambda_{TFBG}cos\theta_{TFBG},$ where *Λ_TFBG_* represents the grating period along the axis fiber.

TFBG structures have also been employed to monitor the magnetic field. For instance, Childs et al. proposed a two 3.2° TFBGs based ring resonance cavity structure, which was encapsulated into a silica capillary filled with EMG 605 MF, for transverse magnetic field monitoring [37]. The sensing principle is based on the reduction of the fringe visibility of the interference on the ghost mode. The sensor exhibited a strong azimuthal dependence on the applied field, and a good linearity over the range of 0.03 to 0.14 T, being the achieved accuracy of 1.4 × 10^−3^ T. Since the resonance structure is based on the ghost mode, the difficulty to fabricate the sensor and the production cost are point out as the main disadvantages of the present sensor.

**Figure 5:**
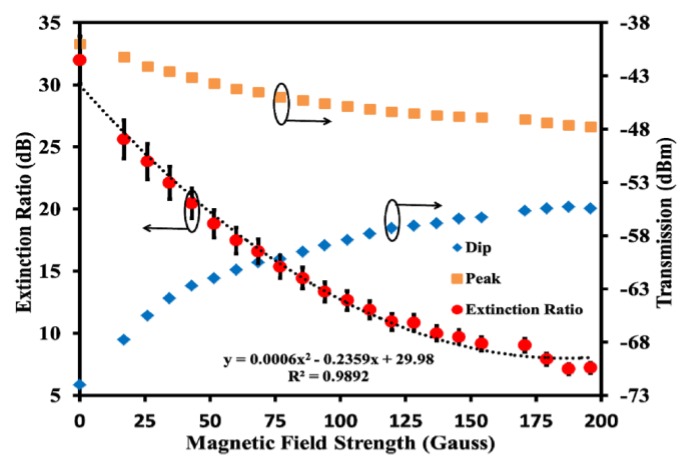
Dependence of the extinction ratio with the magnetic field. Reproduced from [38], with the permission of OSA.

In the case of the work proposed by Zheng et al., a 2° TFBG inserted into an EMG 605 MF-filled capillary was used for the simultaneous measurement of the magnetic field and temperature [38]. The first parameter is determined by detecting variations of extinction ratio of the cladding modes resonance, being the extinction ratio defined as the difference between the peak and the dip powers in the transmission spectrum (see Figure 5), and the later by detecting the wavelength shift of the transmission spectrum. With the proposed structure, magnetic field strength up to 196 G was successfully measured, when this was applied perpendicularly to the fiber axis, being the thermal sensitivity of 8.4 pm/°C.

**Figure 6:**
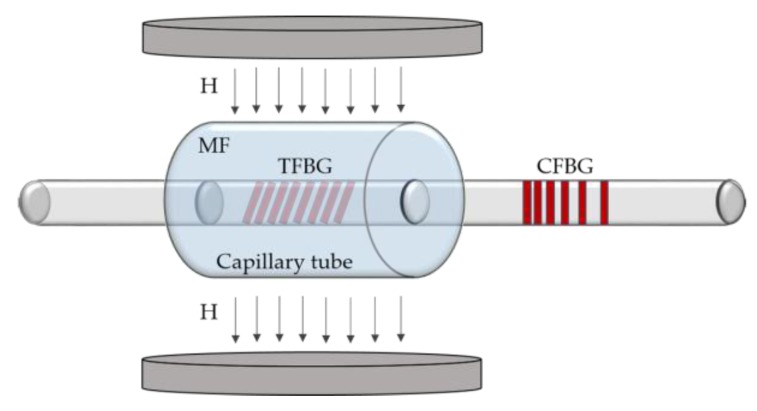
Experimental setup for the magnetic field measurement based on a CFBG-cascaded TFBG (adapted from [39]).

**Figure 7:**
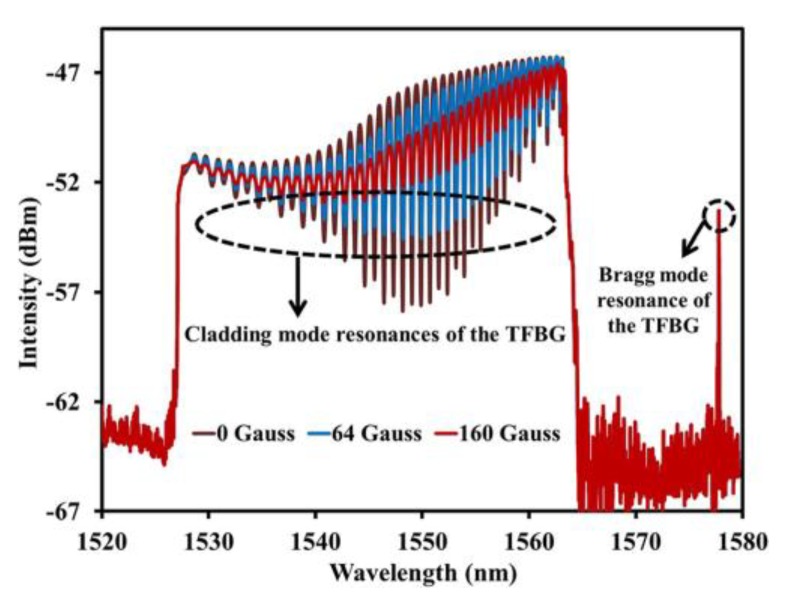
Reflection spectrum of the CFBG-cascaded TFBG structure for different magnetic field intensities. Reproduced from [39], with the permission of AIP Publishing.

Right after, the same research group proposed another sensor, in this case based on an EMG 605-coated 6° TFBG, cascaded by a chirped FBG (CFBG), as schematized in Figure 6 [39]. With this configuration, the optical signal is reflected and modulated twice, which improves the sensor sensitivity. By applying a magnetic field perpendicularly to the fiber axis and increasing its strength from 0 to 196 G, a sharp decrease of the reflected optical power is obtained for low strengths (less than 80 G), being the sensitivity of 147 nW/G. Figure 7 shows the evolution of the reflection spectrum of the CFBG-cascaded TFBG structure with the magnetic field.

Yang et al. used a 10° TFBG inserted into a capillary filled with EMG 705 MF. The magnetic field, induced by an electromagnet in the range of 0 to 32 mT, was applied perpendicularly to the axis of the TFBG [40]. A linearly and sharply blue wavelength shift of the cladding modes was obtained from 0 to 19 mT, and additionally, no hysteresis effect was verified in the sensor response. Comparing with other fiber grating-based solutions, the TFBG-magnetic field sensors have the merit to enable the temperature compensation or the simultaneous measurement of magnetic field and temperature by monitoring the Bragg wavelength shift of the core mode, as already previously discussed.

### 3.3. LPG-Based Sensors

**Figure 8:**
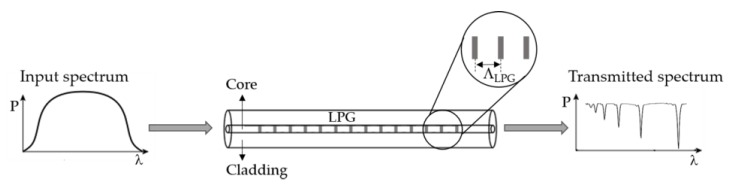
Schematic representation of a LPG.

In an LPG, the periodicity of the refractive index modulation is typically in the range of 100 μm to 1 mm, instead of ≈ 0.5 μm as the FBG and TFBG. Consequently, the light coupling occurs between the core mode and the co-propagating cladding modes, with the LPG acting as a spectral loss selection. In the LPG, the cladding modes are quickly attenuated due to the scattering losses at the cladding-air interface, and consequently, the transmission spectrum of an LPG has several loss bands, at different wavelengths (see Figure 8), given by [41]:

(5) $$\lambda_{i}=\left(n_{core}-n_{clad}^{i}\right)\Lambda_{LPG}$$

where $\lambda_{i}\;$ is the center wavelength of the *i^th^* attenuation band, and $n_{core}$ and $n_{clad}^{i}$ are the effective refractive indices of the core mode and the *i^th^* cladding mode, respectively. $\Lambda_{LPG}$ represents the grating period.

**Figure 9:**
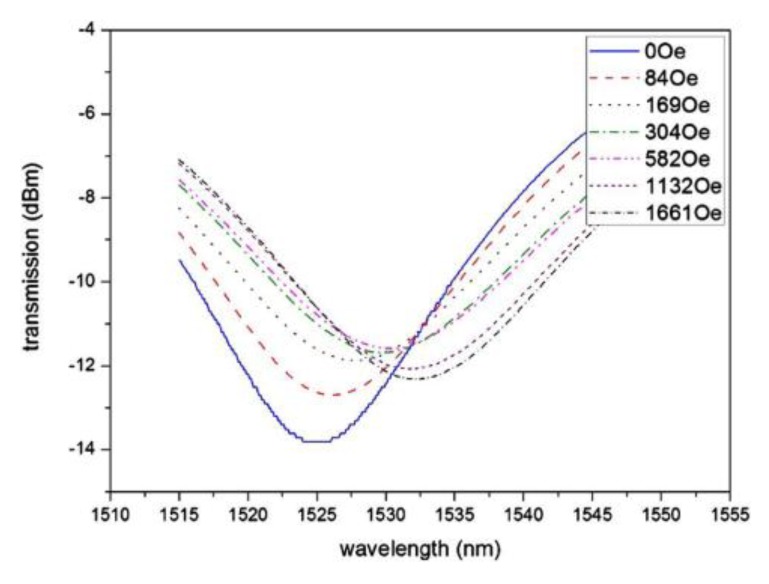
Transmission spectrum of the LPG-based tunable optical filter under different magnetic field intensities. Reproduced from [29], with the permission of AIP Publishing.

**Figure 10:**
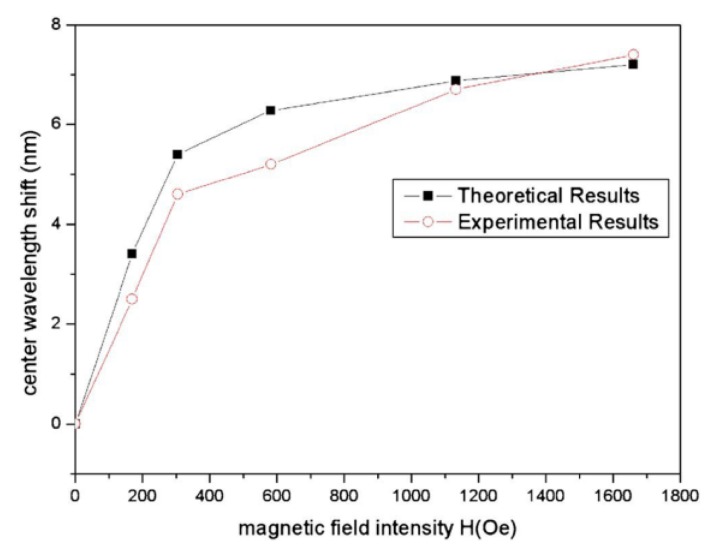
Theoretical and experimental center wavelength shift dependence with the magnetic field. Reproduced from [29], with the permission of AIP Publishing.

Using an LPG surrounded by Fe_3_O_4_ MF, Liu et al. proposed a tunable optical filter whose working principle is based on the tuning of the coupling between the core mode and the cladding modes, through the application of a magnetic field, in this case perpendicular to the LPG [29]. In other words, by changing the refractive index of the MF through the magneto-optical effect, the center wavelength of the attenuation bands of the LPG can be turned, as shown in Figure 9. The decrease of the depth of the attenuation band obtained with the increase of the magnetic field is attributed to the differences in the absorption of the MF under the magnetic field. A center wavelength shift of 7.4 nm is obtained when the magnetic field increases up to 1661 Oe (see Figure 10). This tuning range can be changed and improved depending on the magnetic particles and the carriers of the MF chosen. 

In the work proposed by Miao et al., an LPG was inscribed into a micro-structured optical fiber (MOF) using a scanning CO_2_ laser, and the fiber air holes were filled with EMG 605 MF [42]. This structure was characterized to a variable magnetic field, applied perpendicularly, in the range of 0 to 1661 Oe. There is an increase of the resonance wavelength with the magnetic field, more pronounced up to 300 Oe. In this restricted range, a sensitivity coefficient of 1.946 nm/Oe was obtained.

In [43], the simultaneous measurement of the magnetic field and temperature is proposed, using an LPG surrounded by Fe_3_O_4_ MF. The sensing principle of the sensor is based in the fact that both the wavelength and intensity of the spectral resonance are sensitive to magnetic field and temperature variations. Using the two-parameter matrix method, it is possible to overcome the cross-sensitivity issue. Due to the saturation of magnetic nanoparticle agglomeration within the MF, the proposed sensor may not be suitable for high magnetic field sensing. Nevertheless, the main advantages point out are the low-cost, compactness and temperature cross-sensitivity.

## 4. Interferometric Sensors

### 4.1. FPI-Based Sensors

**Figure 11:**
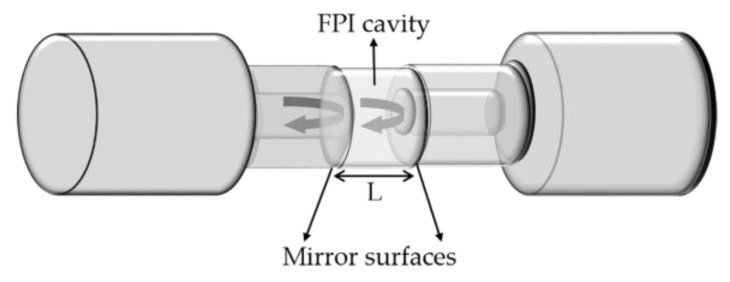
Schematic representation of a FPI.

An FPI is characterized by two reflecting surfaces, delimiting a cavity, with a given intermediate medium, as shown in Figure 11. The latter creates a periodic reflection in the spectral frequency domain, being the phase of the reflected optical signal (*φ*_FPI_) given by the following equation [44]:

(6) $$\emptyset_{FPI}=\frac{4\pi }{\lambda }nL_{FPI}$$

where *λ* is the optical signal wavelength, *n* is the refractive index of the material present in the cavity and *L_FPI_* is the cavity length.

The working principle of this type of sensor can be described based on the dependence of the spectral modulation period (*Λ_FPI_*) with the refractive index of the cavity’s material and its physical length, according with the following equation:

(7) $$\Lambda_{FPI}=\frac{\lambda^{2}}{2nL_{FPI}}$$

FPI cavities-based sensors have become one of the most attractive sensing schemes, due to linear response, sensitivity and reduced dimensions. Normally, the cavity production involves the use of costly technology, for example a pulsed femtosecond laser, with a complex optical alignment and positioning system [45]. However, a cost-effective process was proposed, which was based on the recycling of the optical fiber destroyed by the catastrophic fuse effect, for cavities production [46,47,48].

Zhao et al. used a hollow-core photonic crystal fiber (HC-PCF), with the central core filled with CdFe_2_O_4_ and working as sensitive medium of the FPI, to develop a magnetic field sensor [49]. When the intensity of the magnetic field increases, the optical path difference varies with the increase of the MF’s refractive index, and then, a shift to a longer wavelength direction in the modulation spectrum is verified. To improve the reflectivity of the two interfaces and enhance the power of the signal, the coating technology (using Ti_2_O_3_) and the reflection mirror were adopted. A sensitivity of about 33 pm/Oe was obtained, in the measurement range from 50 to 150 Oe. Since the HC-PCF is insensitive to the environmental temperature, the influence of this parameter in the measurements is neglected. However, the main disadvantages of this sensor are the high HC-PCF cost and the absence of maturation of the fusion technique between the PCF and a single mode fiber (SMF).

The magnetic field sensor described in [50] is constituted by two SMFs and a glass capillary tube. First, one end of the fiber was inserted into the tube, then the tube was filled with EMG 507 MF, and finally the second fiber was inserted into the other side of the tube. The fibers were fixed with UV glue. The separation between the two SMFs, corresponding to the cavity length, was of 32 μm. In the measurement range from 0 to 400 G, a linear dependence of the spectrum shift with the applied magnetic field was obtained, achieving a sensitivity and a resolution of 0.0431 nm/G and 0.5 G, respectively. Compared with other devices, many favorable features were point out to this sensor, including simple structure, compact, low cost, high sensitivity and easy fabrication. However, the main drawback is the thermal effect on the MF’s refractive index which is not considered. 

**Figure 12:**
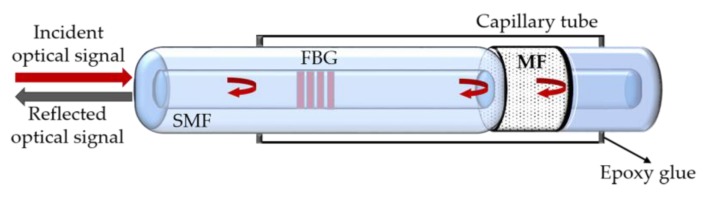
Schematic representation of a FPI based magnetic field sensor (adapted from [51]).

Later, it was proposed a modified sensor structure to overcome the cross-sensitivity effect of the temperature and magnetic field in the MF [51]. This improved sensor version includes a FBG inscribed in the insert fiber end of the FPI cavity (cavity length of 36 μm) which is sensitive to temperature variations, but insensitive to magnetic field changes (see Figure 12). Since no coating film was used on the fiber end faces to improve the reflectivity, an FBG with also low reflectivity was used. With the proposed probe, a sensitivity of 0.04 nm/G and a resolution of 0.5 G were achieved (considering an optical spectrum analyzer resolution of 20 pm), for a magnetic field intensity up to 600 G. The temperature measurement sensitivity is 0.02 nm/°C.

**Figure 13:**
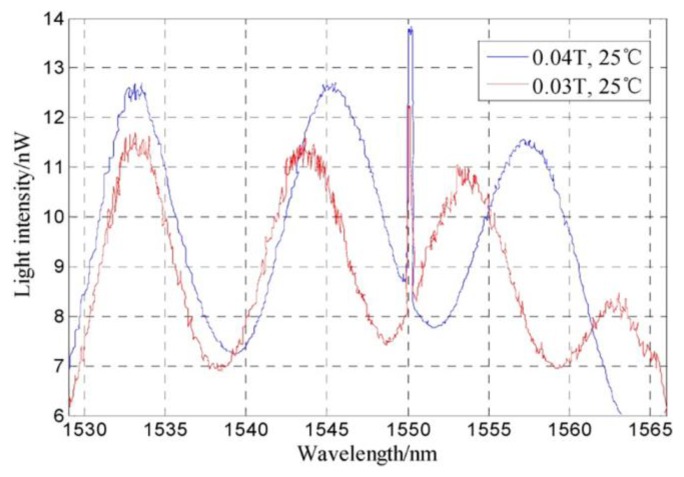
Optical reflection spectra of the FPI-FBG based sensor under two magnetic fields. Reproduced from [52].

**Figure 14:**
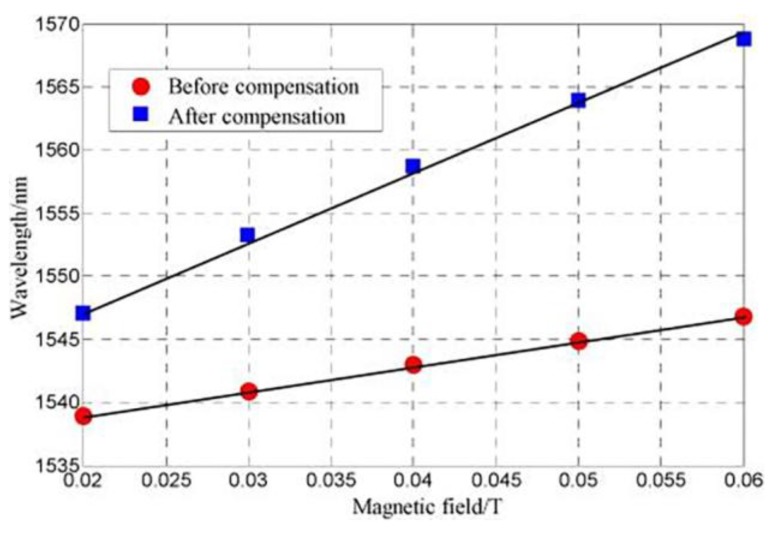
Comparison of the magnetic field sensitivity before and after thermal effect compensation. Reproduced from [52].

Although the sensor previously described in [51] already allowed the simultaneous temperature and magnetic field monitoring, Xia et al. presented a study where the influence of temperature on the sensor sensitivity is experimentally assessed [52]. Figure 13 shows the spectrum of the FPI-FBG structure under two magnetic fields. An improvement on the sensitivity on the magnetic field detection from 0.23 nm/mT to 0.53 nm/mT was obtained, when the temperature effect is compensated (see Figure 14). Additionally, the measurement resolution could reach 37.7 μT. The configuration of the sensor is similar to the one used in [51]. 

### 4.2. MZI-Based Sensors

MZI basic configuration consists in two independent optical paths, which are the reference and the sensing path. The optical signal injected in the optical fiber is split into both paths (arms), recoupled, and modulated according to the optical path difference (OPD) between the two different arms. The interferometric signal created by the recombination of the two signals in the different paths will reflect the external influences of the parameter to monitor, in the sensing arm. As the reference arm should be isolated from any interaction with environment, the interferometric shift in the final optical signal will be only due to the influence of the parameters (strain, refractive index, temperature or other), in the optical signal of the sensing arms. 

**Figure 15:**
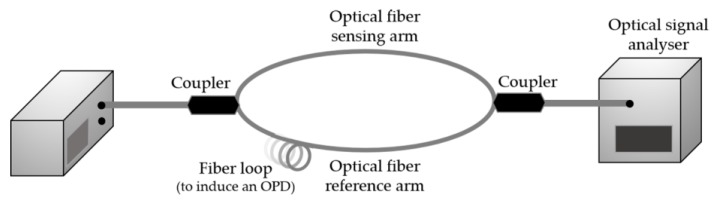
Experimental diagram of an MZI based on two optical fibers with different lengths.

The different paths needed for this type of sensor can be achieved by using two optical fibers with different lengths, as schematically represented in Figure 15, or only one optical fiber, as for the in-line MZI. In the later, both optical paths have the same physical length, but, in such configuration, the OPD is created by the difference in the effective refractive index between the optical fiber core and the cladding, which induces a modal dispersion of the optical signal. Among the techniques used to produce this type of in-line MZI, there are the use of LPGs in SMF, the production of a mismatch in the fiber core or even the use of optical fibers with different fiber core diameters [53,54].

Zu et al. proposed a magnetic field sensor combining MF and MZI operated based on modal interference of two collapsed points of air-hole cladding on the PCF length [55]. The collapsed PCF was immersed into EMG 605 MF, and submitted to an external magnetic field, applied perpendicularly to the MF covered PCF. Consequently, the effective refractive indices of the cladding modes change, while of the core mode is unaffected. This results in a difference in the refractive index, as well as a change in the phase. Therefore, a transmission spectrum shift with the increase of the external magnetic field strength is obtained. With the proposed sensor, a sensitivity and a resolution of 2.367 pm/Oe and 4.22 Oe were achieved, respectively, for a magnetic field strength up to 0.3 kOe. Additionally, no hysteresis was found in the sensor response, and due to the PCF-ultralow temperature coefficient (3.2 pm/°C), the sensor can be considered thermal-insensitive.

**Figure 16:**
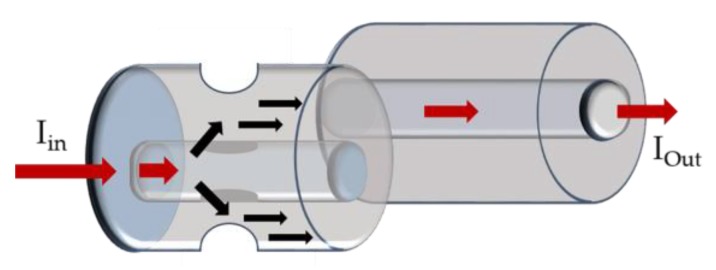
Schematic diagram of an MZI based on a tapered fiber cascaded with a core-offset structure (adapted from [56]).

The sensor proposed in [56] is based on an asymmetrical fiber modal MZI, obtained by cascading tapered SMF with a core-offset structure, which is immersed into EMG 605 MF, using a glass capillary tube (see Figure 16). As the magnetic field varies, the refractive index of the MF changes, inducing variations of the effective refractive index and the propagation constants of the cladding modes. Therefore, the resulting transmission power variation can be used to monitor magnetic field. The sensor was subjected to a magnetic field range from 0 to 400 Oe, generated by two magnets, and applied perpendicular to the fiber axis. A linear decrease of the transmission power with the magnetic field intensity was obtained for the range 100–300 Oe, being the maximum sensitivity achieved of −0.03407 dB/Oe. The compact size, robustness, ease of fabrication and low cost are the advantages point out to the device.

A modal MZI based on a MF-filled PCF is presented in [57]. The MZ interference is induced by a SMF-PCF-SMF structure, where multiple cladding modes are involved in the interference pattern. These modes present different sensing sensitivities, hence, simultaneous measurement of magnetic field and temperature can be accomplished by monitoring the wavelength shift of different modes in the interference spectra. Note that the high order modes have more sensitivity to these parameters than lower order modes and the core mode. The wavelength shift of the spectra dips is linearly proportional to the applied magnetic field and temperature, providing a magnetic field sensitivity of 0.072 nm/G, in the range 0–66.6 G, and a high temperature sensitivity of −0.080 nm/°C. The described sensor presents a set of advantages, highlighting the overcoming of the temperature cross-sensitivity problem.

### 4.3. MI-Based Sensors

**Figure 17:**
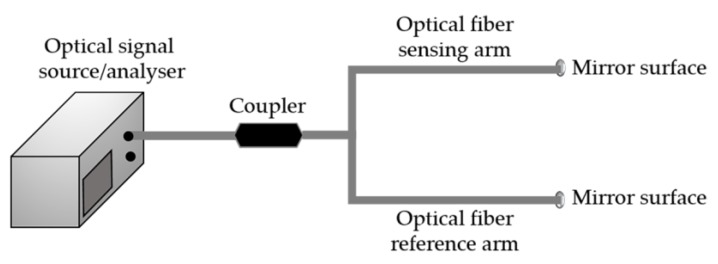
Schematic diagram of a MI experimental setup.

Similar to the MZI, also MI needs two arms to produce an interferometric optical signal (the sensing arm and the reference arm). However, opposite to an MZI configuration, the optical signal propagating in each arm is reflected at the end tip, and recoupled again into the same fiber, generating an interference pattern dependent also of the OPD difference induced by the parameters to be monitored. Figure 17 holds a schematic diagram of a MI [58,59]. An in-line configuration of the MI can be achieved by coupling the core mode into the cladding, by using, for example, LPGs. In such configuration, both cladding and core modes can be reflected by a mirror surface at the fiber end tip, and then recoupled back into the fiber core [58,59,60].

**Figure 18:**
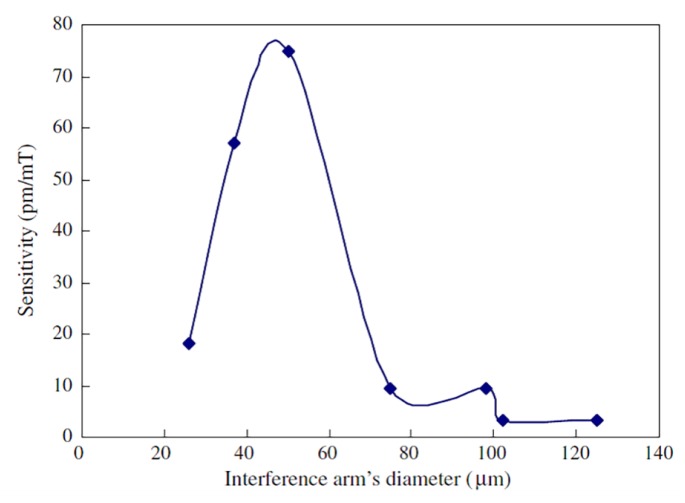
Correlation between the interference arm’s diameter and the magnetic field sensitivity. Reproduced from [61], with the permission of OSA.

Deng et al. proposed an in-line optical fiber MI-based magnetic field sensor, with Fe_3_O_4_ MF acting as the modified cladding [61]. The MI was produced in a SMF by a high frequency CO_2_ laser, which creates visible damages on the fiber surface and made an unilateral microstructured shape, whose notch depth is around 60 μm. When the light beam reaches the air notch, the mode field diameter of the core mode enlarges, and part of the optical signal is coupled into the cladding. Due to the mode-field mismatch, the cladding modes are excited. Therefore, at the notch, the input optical beam is split into two optical paths, which are reflected at the end face of the fiber. The reflected optical signal is then recombined and interfere at the notch, leading to smooth and regular interference fringe with relatively high contrast. Since the MI was immersed into the MF (except the notch and the end face of the SMF), the asymmetric cladding mode interact with the MF as evanescent field, while the core mode is not affected. Thus, as consequence of the variation of the magnetic field intensity, the refractive index of the MF changes, which is detectable by the reflected spectrum shift. Aiming to improve the performance of the proposed sensor, the wet-chemical etching method was used to reduce the diameter of the interference arm. The sensitivity of the sensors with different arm’s diameters (125–20 μm) was experimental investigated and the results reveal that the sensor is most sensitive to the external magnetic field when the arm’s diameter is 50 μm (see Figure 18). For magnetic field strength up to 200 mT and applied perpendicularly to the sensor, the sensitivity found was 64.9 pm/mT, which is 20 times higher than that of 125 μm. The ease, simplicity and time-saving are the advantages point out to this sensor. 

### 4.4. SI-Based Sensors

**Figure 19:**
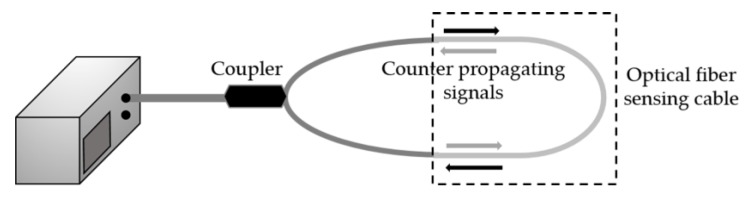
Schematic diagram of a SI experimental setup.

In comparison with the previous configurations, SI is considerably easier to produce. Its setup consists of an optical fiber loop in which two optical signals with different polarizations are propagating in opposite directions. Figure 19 is a schematic representation of a SI layout. The optical signal, generated at the optical source, is split in two. The two signals counter propagating are then recoupled again in the same fiber, generating an interferometric pattern, as a result of the interference between the signals polarized along the fast and the slow axis. The SI phase (*φ*_SI_) can be written as:

(8) $$\emptyset_{SI}=\frac{2\pi }{\lambda }BL_{SI}\;with\;B=\left|n_{fast}-n_{slow}\right|$$

where *L_SI_* corresponds to the length of the polarized sensing path, *B* is the birefringent coefficient, and *n_fast_* and *n_slow_* are the effective refractive indices of the fast and slow modes, respectively [54]. 

The SI monitoring is based on the OPD induced by polarization dependent speed of the guided modes along the loop, which can be enhanced by using birefringent optical fibers [62]. Due to the high thermal expansion coefficient of these fibers, the sensing characteristics of SIs can be used for high precision temperature monitoring [63,64]. 

**Figure 20:**
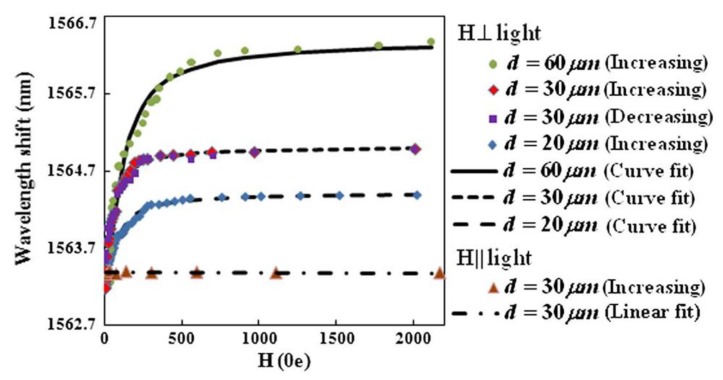
Dependence of the dip wavelength with the magnetic field, for perpendicular and parallel cases. Reproduced from [65], with the permission of OSA.

In [65], a magnetic field sensor based on the birefringence property of the MF is demonstrated. Until now, in the later reported sensing configurations, only the tunable refractive index effect of the MF was used in the most MF-based magnetic field sensors. However, since the thickness of the MF film is so small that cannot generate a phase difference enough to be measured, a small section of polarization maintaining fiber (PMF) was inserted into the Sagnac loop, to provide an initial constant phase difference. The performance of the sensor was assessed in terms of the magnetic field direction, and also the MF film thickness. For the parallel case, both the dip wavelength and extinction ratio of the transmission spectrum remain almost constant, as the magnetic field strength increases. In this case, the Faraday effect is considered, while birefringence and dichroism properties are neglected. For the perpendicular case, both birefringence and dichroism properties are considered, while Faraday effect is ignored, and a spectrum shift to longer wavelengths is observed when the magnetic field strength increases from 0 to 2013 Oe. The extinction ratio also decreases due to the dichroism effect of the MF film. The best sensitivity and resolution were obtained for the thickest film tested (60 μm), namely 16.7 pm/Oe and 0.60 Oe, respectively, for the range 0 to 300 Oe (see Figure 20). Additionally, the device was also assessed as dynamic magnetic field sensor. In the frequency response range of about 100 Hz, a sensitivity of 0.3998 dB/Oe was obtained, with a response time of 10–30 ms and a good repeatability. 

**Figure 21:**
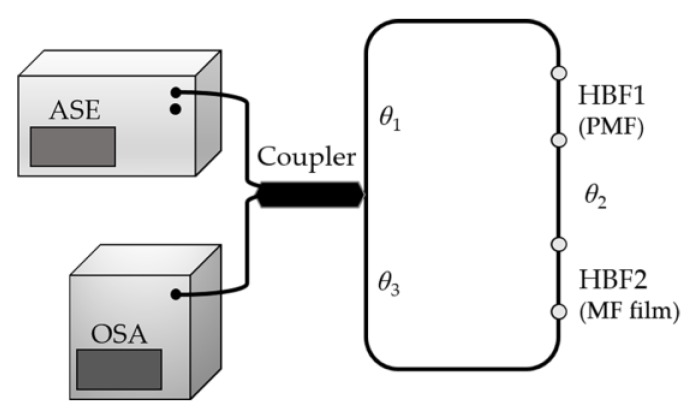
Schematic diagram of a Loyt–Sagnac interferometer (adapted from [66]).

Later, the same research group proposed another sensor, based on a Loyt–Sagnac interferometer configuration with an enhanced sensitivity [66]. In this case, there is PMF in the position of the high birefringence fiber (HBF), HBF1, while the MF film is located in HBF2. Figure 21 shows a schematically representation of this configuration, with *θ*_1_ and *θ*_3_ representing the angles between the polarization directions of counterpropagating beams and the corresponding fast axes of the two HBF sections, and *θ*_2_ is the angle between the fast axes of the two HBF sections. The predicted theoretical behaviors were experimentally verified. For this, PMF with different lengths (300, 50 and 2 cm) and MF film with different thickness (20, 40, 70 μm) were tested. As expected, the sensor sensitivity improves with the increasing of the film thickness and decreases with the PMF length. The best sensitivity, in this case 592.8 pm/Oe (0–200 Oe), was obtained with 2 cm-PMF length and a MF film with 70 μm. 

Zhao et al. proposed a sensor whose sensing element is a ferrofluid-filled HB-PCF, which was inserted into a Sagnac loop [67]. The working principle is based on the change of the refractive index of the MF (EMG 507) with the applied magnetic field, which modifies the birefringence of the high birefringence PCF (HB-PCF), detectable through a shift of the output interference spectrum in the SI. A simulation model based on the full-vector finite element method (FEM) was used to study the HB-PCF birefringence property. The sensor sensitivity is up to 1.073 nm/mT and the resolution is 0.001 mT, for a magnetic field ranging from 10 to 40 mT. The difference between the experimental and theoretical (1.180 nm/mT) sensitivity values is attributed to the thermal variation that occurs when the current flowed through the coils (in this case the magnetic field is applied parallel to the light path direction), which affects the MF’s refractive index, and in this case was not compensated. To improve the measurement range of the sensor, the authors point out three possible procedures, namely the use of MF with a high saturation magnetization, HF-PCF length shortening, and HB-PCF with higher birefringence.

## 5. Other Sensing Schemes Based Sensors

### 5.1. SPR-Based Sensors

**Figure 22:**
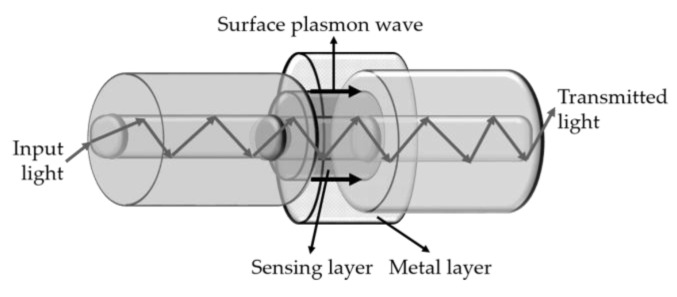
Schematic diagram of a typical optical fiber SPR-based sensor.

The SPR sensing principle is based on an optical phenomenon in which a TM-polarized light beam satisfies the resonance condition and excites the free electron density longitudinal oscillation at the metal/dielectric interface [68,69]. This resonance condition is dependent of distinct parameters, including incident angle, metal dielectric constant and incident light wavelength. The metals most frequently used to induce the SPR phenomenon are gold and silver. Figure 22 is a schematic diagram of a typical optical fiber SPR-based sensor. 

Optical fiber SPR-based sensors are recognized for its high refractive index sensitivity, label free and real-time detection. It has been applied to many fields, including food safety, healthcare, environment monitoring [70,71,72] and magnetic field detection, as discussed below. 

The sensor proposed in [73] consists in a doubly-deposited uniform waist tapered fiber (DLUWT) SPR fiber optic sensor, combined with EMG 607 MF. This double deposition, whose function is to enable the plasmonic resonance wavelength tuning, comprises 19 nm of aluminum and 60 nm of titanium dioxide (60 nm). The taper dimensions were: 40 μm of waist diameter, 6.34 mm of waist length and 28.30 mm of total length. A magnetic field (up to 10 mT) was applied parallel to the fiber axis, and a sensitivity and resolution around 10 nm/mT and 0.3 mT (linear behavior) were achieved, respectively. The influence of the temperature increase resulting from the coils heating, and the hysteresis effect were investigated, being neglected its impact.

**Figure 23:**
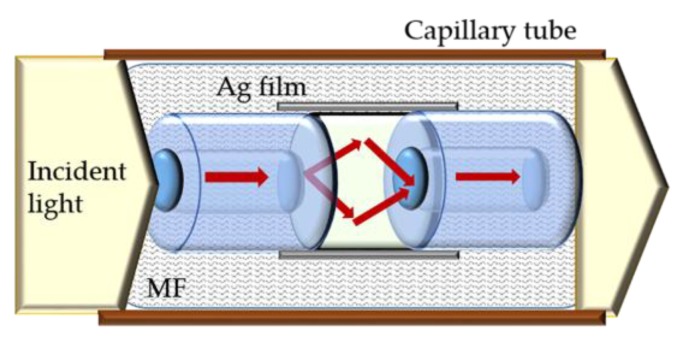
Schematic diagram of a magnetic field sensor based on a MNF structure (adapted from [74]).

A magnetic field sensor based on SPR optical fiber interacting with EMG 507 is also proposed in [74]. The sensor comprises a no-core fiber fused between two multimode fibers (MNF), coated with an Ag film (see Figure 23). The sensitivity of the designed sensor is up to 303 pm/G, in the range from 0 to 349 G. The accuracy of the device was also assessed, increasing and decreasing the magnetic field. There was a slight difference in the obtained values, attributed to temperature effects. The simple structure, small volume, easy to make and low cost are the main advantages pointed out to the sensor.

Liu et al. simulated a temperature compensated magnetic field sensor based on a D-shaped and MF-infiltrated PCF, and the SPR phenomenon [75]. The temperature induced effect was eliminated by inserting a defective cavity filled with toluene solution in the cladding layer, and an Au–Ag film was the composite used as coating material, instead of a traditional gold layer, aiming to reduce the loss. The sensing characteristics of the proposed sensor was investigated using the FEM and perfectly matched layer (PML) boundary conditions. A sensitivity of 0.87 nm/mT was estimated for a magnetic field in the range of 0 to 55 mT. 

### 5.2. Etched, Tapered, U-Shaped Fibers

Due to the low interaction of the core mode/lower cladding modes and the MF, several sensors above discussed present sensitivities lower than the desired values. The etching and tapering of the fiber in the sensing region and the bending of the fiber into an U-shaped are, for instance, procedures which can be carried out to enhance the evanescent field effect in the sensing structure [76,77].

Wang et al. developed a magnetic field sensor based on a fiber loop ring-down spectroscopy (FLRDS) and etched fiber (cladding diameter of 18.83 μm) interacting with EMG 507 MF [78]. Both the magnetic tunable refractive index and absorption coefficient transmission properties of the MF were simultaneously considered, and the transmission spectrum is modulated by the FLRDS, which improves significantly the sensitivity and the anti-interference. When a magnetic field is applied perpendicularly to the light propagation, a sensitivity of −12.56 G/μs and a resolution of 25 G were achieved, for magnetic fields up to 650 G. Although the etching treatment improves the sensor sensitivity, it also triggers a relatively large inherent loss on the fiber loop, which affects negatively the FLRDS accuracy and compromises the mechanical robustness of the device.

**Figure 24:**
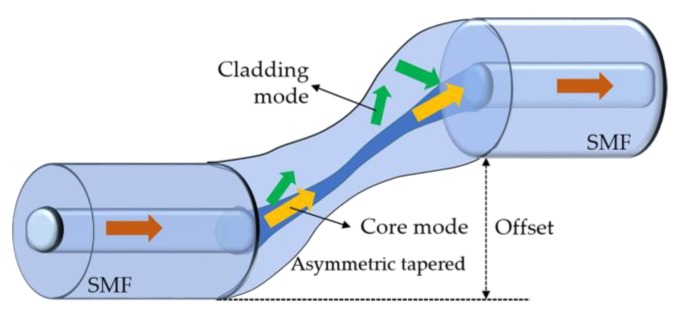
Schematic diagram of the CCMI based sensor (adapted from [79]).

The sensor proposed in [79] consists in a core cladding mode interferometer (CCMI) based on an asymmetric taper (see Figure 24). This was produced on a SMF with a Furukawa S176 fusion splicing machine, using multiple arc discharges with low power. The input optical beam is split into two optical paths at the upper taper, along the SMF core and cladding, and after transmitting a short distance, they are recombined and interfere at the down taper, leading to a smooth and regular interference fringe. A CCMI with axial offset of 168 μm and a taper waist diameter of 45 μm was inserted into a glass capillary, filled with Fe_3_O_4_ MF, and subjected to a magnetic field, applied perpendicularly. When the magnetic field ranges from 0 to 185.2 mT, a significant decrease of the intensity is obtained, with a sensitivity of −0.098 dB/mT. This is result of the scattering effect caused by the evanescent field of the cladding modes propagating throughout the metal particles on the optical fiber surface. When the magnetic field is smaller than 21.4 mT, a sharp dip wavelength shift is verified, with a sensitivity of about −162.06 pm/mT. The sensor’s hysteresis was also investigated, and although this results from the magnetic fluid viscosity, this problem could be minimized if the magnetic field was changed more slowly. Additionally, the intensity and the wavelength demodulation methods were compared, with the last one leading to the smallest errors. The authors also compared the sensor’s performance produced with different parameters, namely axial offset of 142 and 113 μm and taper waist diameter of 44 and 43 μm, respectively, concluding that the magnetic field sensitivity increases with the taper’s axial offset, since higher cladding modes are excited at the upper taper. The sensor was considered thermal insensitive in the range from 20 to 100 °C, since the thermal coefficients of the optical fiber and the MF are positive and negative, respectively.

**Figure 25:**
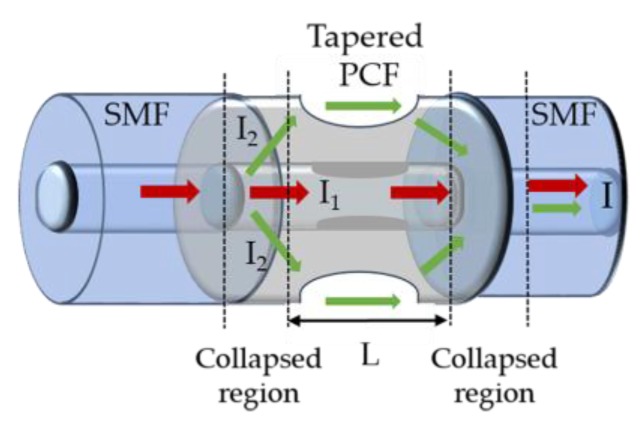
Schematic diagram of the magnetic field sensor based on a tapered PCF (adapted from [80]).

Zhao et al. proposed a magnetic field which consists of a tapered PCF with a waist diameter of 24 μm and a length of 804 μm, spliced between two SMFs, and coated with EMG 507 MF (see Figure 25) [80]. In this sensing structure (MZI), a strong evanescent field was produced near the tapered region, with sensitivity to refractive index variations, and consequently to magnetic field changes. Therefore, as a magnetic field is applied parallel to the fiber axis, in the range from 100 to 600 G, a shift in the interference spectrum is obtained with a sensitivity of 16.04 pm/G and a resolution of 0.62 G. Due to the hysteresis time of the MF, the sensor’s response is 30 min. The main advantages of the proposed sensor are its compactness, high sensitivity, robustness and easy fabrication.

In [81], it is proposed a magnetic field sensor based on a single mode-multi mode-single mode (SMS) fiber structure, with a no-core fiber (NCF) sandwiched between two SMFs and coated with EMG 605 MF. The performance of the straight sensor was compared with the case where the NCF with a diameter of 61.5 μm is U-bent, being the curvature radius of 2.5 cm. Although the changes in the transmission spectra are similar for the two sensor configurations, the variations of the dip wavelength and the transmission loss are more accentuated for the U-bent sensor. A sensitivity of 3185.2 pm/mT was obtained when a magnetic field was perpendicularly applied in the range of 1.6 to 9.6 mT. This is 4.3 times larger than the value obtained for the straight sensor. The effect of the NCF diameter in the sensor’s performance was also investigated and, as expected, smaller diameter results in a higher sensitivity. Additionally, from the experimental tests, the authors considered that the errors induced by the thermal sensitivity can be neglected in common applications. 

**Figure 26:**
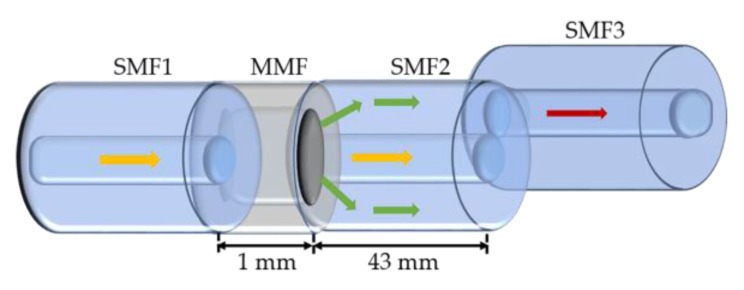
Schematic diagram of a magnetic field sensor based on an SMS fiber cascaded structure with core-offset fusion splicing between SMFs (adapted from [82]).

Luo et al. also proposed a magnetic field sensor based on SMS fiber cascaded structure with core-offset fusion between SMFs, whose sensing principle is based on the cladding mode interference (see Figure 26) [82]. The sensor structure was positioned into a capillary, filled with Fe_3_O_4_ MF, and subjected to a magnetic field applied perpendicularly to the optical fiber axis. The experimental results reveal an increase of the wavelength and the intensity of the interference dip when the magnetic field changes from 0 to 110 Oe. The dip becomes imperceptible above this value. A magnetic field sensing sensitivity of 65.9 pm/Oe and 0.1185 dB/Oe was obtained for wavelength and intensity demodulations, respectively, for the range of 30 to 110 Oe. Applying magnetic field at ascending and descending orders for several cycles, the hysteresis effect on the sensor response was assessed. Additionally, the thermal response was also investigated, and sensitivities of −42 pm/°C and −0.1244 dB/°C were obtained for the wavelength and transmission loss demodulations. To overcome the cross-sensitivity of these two parameters, the two-parameter matrix method could be applied. The low-cost, compactness and high-sensitivity are the advantages to point out to this device.

**Figure 27:**
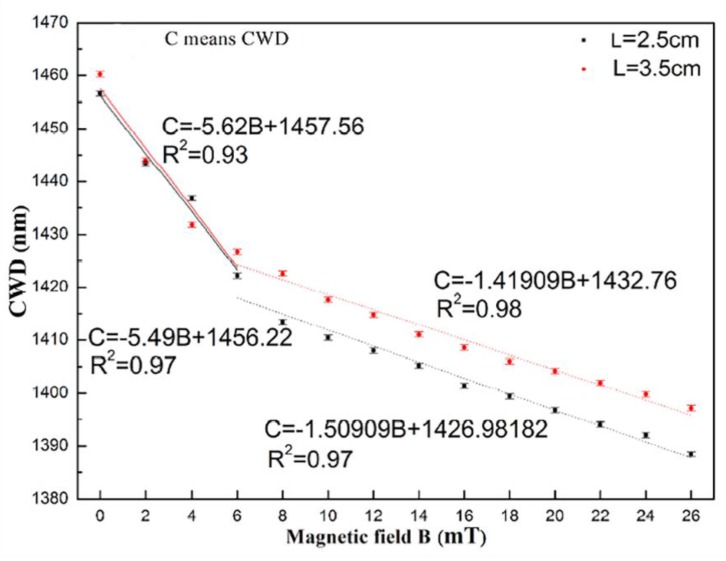
Dependence of the CWD with the magnetic field, for NCF with different lengths [83].

The magnetic field sensor developed by Rao et al. consists in a single mode-no-core-single mode (SNS) fiber structure, which is covered by an oil-based MF (EXP08103) [83]. The sensing principle is based on the refractive index matched coupling (RIMC), used to improve the sensor sensitivity. At RIMC, the guided modes within the NCF become leaky, leading to a coupling wavelength dip (CWD) in the transmission spectrum. Thus, slight variation in the surrounding environment will cause a significant change in the CWD. In this work, two sensing structures, consisting in NCF with lengths of 2.5 and 3.5 cm, were fabricated and tested under a magnetic field applied perpendicularly to the fiber axis, and as shown in the Figure 27, the NCF length has a negligible influence in the sensor sensitivity. 

**Figure 28:**
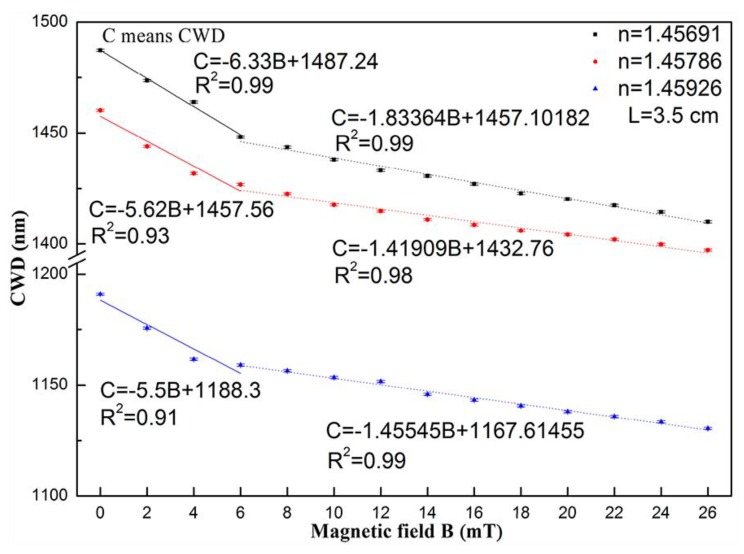
Dependence of the CWD with the magnetic field, for sensing structures covered with MF with distinct refractive indices [83].

The dependence of the CWD with the magnetic field, for the sensing structures, covered with MF with distinct refractive indices, are presented in Figure 28. The highest sensitivities were obtained for the case of the MF with the lowest refractive index, namely 6.33 and 1.83 nm/mT, for lower (≤6 mT) and higher (6–26 mT) magnetic fields, respectively. The maximum sensitivity achieved with this sensor is superior to the values obtained with sensing structures based on similar designs, as is the case of the works presented in [84,85]. Additionally, the sensors based on these structures (SMS, SNS) have the advantage of being low-cost and easy to fabricate.

## 6. Final Remarks

Magnetic fluid is one of the most attractive magneto-optical materials, with a variety of applications in magneto-optical fiber devices, including as magnetic field sensors. These are based on different configurations, such as fiber gratings (FBG, TFBG, LPG, …), light intensity modulation, interferometry, among others. Some examples of optical fiber based magnetic field sensors have been reviewed in the above sections. The combination of the MF with the optical fiber appeared as one of the following ways: as MF film, the filling material and the cladding. The first form was used, for instance, in a Sagnac loop-based sensor [67]. The case of the filling material can be applied to the Fabry–Perot cavities-based sensors [49], and the use of MF as cladding can be demonstrated by the etched/tapered based sensors, which were covered with the MF [79]. For performance comparison purposes, in this table, the characteristics of other sensors reported in the literature are also included.

Table 2 summarizes the main sensing parameters of the MF-based magnetic field sensors discussed previously in this paper. For performance comparison purposes, in this table, the characteristics of other sensors reported in the literature are also included.

From Table 2, it is possible to conclude that the sensing schemes based on LPGs and MIs are the configurations with higher range of magnetic field detection, presenting values up to 166 mT [29] and 200 mT [61], respectively. The highest sensitivity values are obtained using SI, SNS and SPR-based sensors. These designs lead to values around 5928 pm/mT [66], 6330 pm/mT [83] and 10,000 pm/mT [73]. The SI is the sensing scheme that provides the shortest response time, namely 10–30 ms [65], when compared with the 15 s [33] and 30 s [34] reported for the Bragg grating-based sensors. However, this parameter is not often evaluated, since it is limited by the time that the MF takes to reach the equilibrium, after the change of magnetic field intensity [80].

Overall, the superiority of the optical fiber technology together with the versatility and advantages of the MF makes this solution desired over other traditional detection methods, which have made an increasing contribution to the global optical fiber sensors market. 

Nevertheless, and in spite of the advantages referred above, it should be noted that there is still a long way to pave in order to improve the sensors performance and to decrease the production process complexity in determinate cases.

Ongoing research in several fields of engineering applications can be aided by new advances in the field of magnetic sensors, consequently increasing their impact. So, a promising future is foreseen regarding the design and development of optical fiber-based magnetic field sensors and their applications, for instance a particular case, in the electric energy transport circuits (transformation powerplants, high-voltage transport).

## Figures and Tables

**Table 1:** Distribution of the optical fiber sensors discussed in this paper.

Optical Fiber Sensors		
Grating-based sensors	Interferometric based sensors	Other sensing schemes-based sensors
Uniform fiber Bragg grating (FBG) Tilted fiber Bragg grating (TFBG) Long period grating (LPG)	Fabry-Perot interferometer (FPI) Mach-Zehnder interferometer (MZI) Michelson interferometer (MI) Sagnac interferometer (SI)	Surface plasmon resonance (SPR) Tailored fibers (etched, tapered, U-shaped)

**Table 2:** Performance parameters of optical fiber magnetic field sensors based on MF.

Detection Mechanism	Optical Fiber Configuration	Ferrofluid	Magnetic Field Direction (Fiber Axis)	Detecting Range	Sensitivity/Resolution	Response Time	Ref
Wavelength shift	eFBG	Fe_3_O_4_	Perpendicular	0–25 mT	86 pm/25 mT (no linear behavior)	15 s	[33]
Cladding mode intensity	FBG/ TCF	Ferromagnetic particles	Perpendicular	7–15 mT	−0.78 dB/mT	30 s	[34]
Fresnel reflectivity	FBG	EMG 607	Perpendicular	0–5 mT	________________	______	[35]
Wavelength shift	PS-FBG	EMG 605	Perpendicular	0–12 mT	24.2 pm/mT	______	[86]
Fringes visibility Wavelength shift	TFBG	EMG 605	Perpendicular	30–140 mT 30–85 mT	1.4 mT 0.4 pm/mT 2.5 mT	______	[37]
Extinction ratio	TFBG	EMG 605	Perpendicular	0–19.6 mT	________________	______	[38]
Intensity of the reflected optical power	TFBG/CFBG	EMG 605	Perpendicular	0–14 mT	1470 nW/mT (0–8 mT)	______	[39]
Wavelength shift	TFBG	EMG 705	Perpendicular	0–32 mT	106 pm/35 mT (0–19 mT; no linear behavior)	______	[40]
Wavelength shift	LPG	Fe_3_O_4_	Perpendicular	0–166.1 mT	7400 pm/166.1 mT (no linear behavior)	______	[29]
Resonance wavelength shift	LPG (MOF)	EMG 605	Perpendicular	0–166.1 mT	1946 pm/mT (0–30 mT)	______	[42]
Resonance wavelength shift	FPI (HC-PCF)	CdFe_2_O_4_	__________	5–15 mT	330 pm/mT	______	[49]
Resonance wavelength shift	FPI (SMF)	EMG 507	__________	0–40 mT	431 pm/mT 0.05 mT	______	[50]
Resonance wavelength shift	FPI (SMF)/FBG	EMG 507	__________	0–60 mT	400 pm/mT 0.05 mT	______	[51]
Resonance wavelength shift	FPI (SMF)/FBG	EMG 605	Parallel	20–60 mT	530 pm/mT 0.038 mT	______	[52]
Wavelength shift	MZI	EMG 605	Perpendicular	0–30 mT	23.67 pm/mT 0.422 mT	______	[55]
Transmitted power variation	MZI	EMG 605	Perpendicular	0–40 mT	−0.3407 dB/mT (10–30 mT)	______	[56]
Wavelength shift	MZI	EMG 507	Perpendicular	0–6.66 mT	720 pm/mT	______	[57]
Wavelength shift	MI	Fe_3_O_4_	Perpendicular	0–200 mT	64.9 pm/mT	______	[61]
Wavelength shift Intensity variation	SI	EMG 605	Perpendicular	0–30 mT	167 pm/mT 0.060 mT 3.998 dB/mT (dynamic)	10–30 ms	[65]
Wavelength shift	Loyt-SI	EMG 605	Perpendicular	0–20 mT	5928 pm/mT	______	[66]
Wavelength shift	SI	EMG 507	Parallel	10–40 mT	1073 pm/mT 0.001 mT	______	[67]
Wavelength shift	SI	Fe_3_O_4_	Perpendicular	10–25 mT 41–60 mT	5300 and −5800 pm/mT 3840 and −4300 pm/mT	______	[87]
Wavelength shift	SPR/DLUWT	EMG 607	Parallel	<10 mT (only linear behavior)	~10,000 pm/mT ~0.3 mT	______	[73]
Wavelength shift	SPR/MNF	EMG 507	Perpendicular	0–34.9 mT	3030 pm/mT	______	[74]
Wavelength shift	SPR/D-shaped PCF	Fe_3_O_4_		0–55 mT	870 pm/mT	______	[75]
Ring down time	FLRDS/etched fiber	EMG 507	Perpendicular	0–65 mT	−1.256 mT/μs 2.5 mT	______	[78]
Wavelength shift Intensity variation	CCMI	Fe_3_O_4_	Perpendicular	0–21.4 mT 0–185.2 mT	−162.06 pm/mT −0.098 dB/mT	______	[79]
Wavelength shift	Tapered PCF	EMG 507	Parallel	10–60 mT	160.4 pm/mT 0.062 mT	30 min	[80]
Wavelength shift	U-bent NCF	EMG 605	Perpendicular	1.6–9.6 mT	3185.2 pm/mT	______	[81]
Wavelength shift Intensity variation	SMS	Fe_3_O_4_	Perpendicular	3–11 mT	659 pm/mT 1.185 dB/mT	______	[82]
Coupling wavelength dip	SNS/RIMC	Exp08103	Perpendicular	≤6 mT 6–26 mT	6330 pm/mT 1830 pm/mT	______	[83]
Wavelength shift	Etched SMS	Fe_3_O_4_	Perpendicular	12.0–32.5 mT	−168.6 pm/mT	______	[84]
Wavelength shift Transmission loss	SNS	EMG 605	__________	4–10 mT	905 pm/mT 0.748 dB/mT	______	[85]

PS: Phase-shift.

## Appendix A

This appendix contains the acronyms list used in this manuscript.

Acronym	Definition
B	Birefringent coefficient
CCMI	Core cladding mode interferometer
CFBG	Chirped fiber Bragg grating
CWD	Coupling wavelength dip
DLUWT	Doubly-deposited uniform waist tapered fiber
*E*	Electric field
eFBG	Etched fiber Bragg grating
FBG	Uniform fiber Bragg grating
FEM	Finite element method
FLRDS	Fiber loop ring-down spectroscopy
FPI	Fabry-Perot interferometer
*H*	Magnetic field
HBF	High birefringence fiber
HB-PCF	High birefringence photonic crystal fiber
*Hc*	Critical magnetic field
HC-PCF	Hollow-core photonic crystal fiber
*L_FPI_*	Cavity length (FPI)
LPG	Long period grating
*L_SI_*	Length of the polarized sensing path
MF	Magnetic fluid
MI	Michelson interferometer
MMF	Multimode fiber
MNF	No-core fiber fused between two multimode fibers
MOF	Micro-structured optical fiber
*M_s_*	Saturation magnetization
MZI	Mach-Zehnder interferometer
*n*	Refractive index of the material present in the cavity (FPI)
NCF	No-core fiber
*n_core_*	Effective refractive index of the core mode (LPG)
$n_{clad}^{i}$	Effective refractive index of the *i^th^* cladding mode (TFBG)
*n_eff_*	Effective refractive index of the propagate core mode (FBG)
$n_{eff,clad}^{i}$	Refractive index of the *i^th^* cladding mode at $\lambda_{clad}^{i}$ (TFBG)
$n_{eff,core}$	Refractive index of the core mode at *λ_TFBG_* (TFBG)
$n_{eff,core}^{i}$	Refractive index of the core mode at $\lambda_{clad}^{i}$ (TFBG)
*n_fast_*	Effective refractive index of the fast mode (SI)
*n_MF_*	Refractive index of the magnetic fluid
*n_s_*	Saturated value of the refractive index of the MF
*n_slow_*	Effective refractive index of the slow mode (SI)
*n*_0_	Refractive index of the magnetic fluid under magnetic field lower than critical magnetic field
OPD	Optical path difference (MZI)
PCF	Photonic crystal fiber
PMF	Polarization maintaining fiber
PML	Perfectly matched layer
PS	Phase shift
RIMC	Refractive index matched coupling
SI	Sagnac interferometer
SMF	Single mode fiber
SMS	Single mode-multi mode-single mode
SNS	Single mode-no-core-single mode
SPR	Surface plasmon resonance
*T*	Temperature
TCF	Thin core fiber
TFBG	Tilted fiber Bragg grating
UV	Ultraviolet
*α*	Fitting parameter (Langevin function)
*ε_MF_*	Dielectric constant of the magnetic fluid
$\theta_{TFBG}$	Tilt angle of the grating planes related to the perpendicular of the fiber axis (TFBG)
*θ*_1_	Angle between the polarization direction of counterpropagating beams and the corresponding fast axes of two HBF sections (SI)
*θ*_2_	Angle between the fast axes of the two HBF sections (SI)
*θ*_3_	Angle between the polarization direction of counterpropagating beams and the corresponding fast axes of two HBF sections (SI)
*λ*	Optical signal wavelength
*λ_B_*	Reflected Bragg wavelength (FBG)
*λ_i_*	Center wavelength of the *i^th^* attenuation band
λ_TFBG_	Resonance wavelength of the core mode (TFBG)
$\lambda_{clad}^{i}$	Resonance wavelength of the *i^th^* cladding mode (TFBG)
*Λ*	Grating period (FBG)
*Λ_FPI_*	Spectral modulation period (FPI)
$\Lambda_{LPG}$	Grating period (LPG)
*Λ*_TFBG_	Grating period along the axis fiber (TFBG)
*φ_FPI_*	Phase of the reflected optical signal (FPI)
*φ_SI_*	Phase (SI)
*χ*	Electric susceptibility
